# Miniaturized Quantum Semiconductor Surface Plasmon Resonance Platform for Detection of Biological Molecules

**DOI:** 10.3390/bios3020201

**Published:** 2013-06-07

**Authors:** Dominic Lepage, Jan J. Dubowski

**Affiliations:** Laboratory for Quantum Semiconductors and Photon-based BioNanotechnology, Interdisciplinary Institute for Technological Innovation (3IT), Faculty of Engineering, Université de Sherbrooke, 3000 boul. de l’Université, Sherbrooke, QC J1K 0A5, Canada; E-Mail: dominic.lepage@usherbrooke.ca

**Keywords:** surface plasmon resonance, quantum semiconductor emitters, nanophotonic devices, hyperspectral imaging

## Abstract

The concept of a portable, inexpensive and semi-automated biosensing platform, or lab-on-a-chip, is a vision shared by many researchers and venture industries. Under this scope, we have investigated the application of optical emission from quantum well (QW) microstructures for monitoring surface phenomena on gold layers remaining in proximity (<300 nm) with QW microstructures. The uncollimated QW radiation excites surface plasmons (SP) and through the surface plasmon resonance (SPR) effect allows for detection of small perturbation in the density surface adsorbates. The SPR technology is already commonly used for biochemical characterization in pharmaceutical industries, but the reduction of the distance between the SP exciting source and the biosensing platform to a few hundreds of nanometers is an innovative approach enabling us to achieve an ultimate miniaturization of the device. We evaluate the signal quality of this nanophotonic QW-SPR device using hyperspectral-imaging technology, and we compare its performance with that of a standard prism-based commercial system. Two standard biochemical agents are employed for this characterization study: bovine serum albumin and inactivated influenza A virus. With an innovative conical method of SPR data collection, we demonstrate that individually collected SPR scan, each in less than 2.2 s, yield a resolution of the detection at 1.5 × 10^−6^ RIU.

## 1. Introduction

Recent advancements in molecular detection methods, microelectronics, and digital computing have enabled the development of many new tools and applications in biomedical technology. The expansion of innovative biochemical analyses beyond their laboratory environment is still a challenge today, although nanotechnology could present solutions for the development of micro total analysis systems (µTAS) potentially capable of portable biodiagnostics [1]. One of the approaches in that context is based on the surface plasmon resonance (SPR) effect that shows attractive sensitivities to miniscule perturbation in the density surface adsorbates. SPR is a well-established optical phenomenon where an electromagnetic (EM) beam of a specific energy and incident wavevector (angle) can induce a resonant group oscillation within the surface electrons of a metal-dielectric interface [2]. The resulting EM field is evanescent in nature, with typical confinement of 200 nm for visible light and can consequently be employed for many dynamic biochemistry studies [3]. However, the SPR technology has remained in the realm of trained professionals, in academic laboratories and within specialized industries, requiring relatively large space, capital investment and the support of qualified personnel [4]. A Texas Instruments developed SPREETA^TM^ addresses the need of a miniaturized and low-cost device that could help popularize the SPR technology [4]. However, the limited success of this approach could be related to its average performance in terms of sensitivity [5]. 

We present here the results of an effort towards SPR device miniaturization through a wholly integrated semiconductor-based SPR nanometric platform that, at the same time, maintains high sensitivity required for detection of small biological molecules. Our SPR microsystem offers portability, attractive sensitivities, and a drastic reduction in operational costs, consequently opening a potential for delocalized applications. Coupled with integrated electronic circuits, a preset SPR semiconductor chip would ideally be operated by the layperson.

## 2. Experimental Section

### 2.1. Methodology

**Figure 1:**
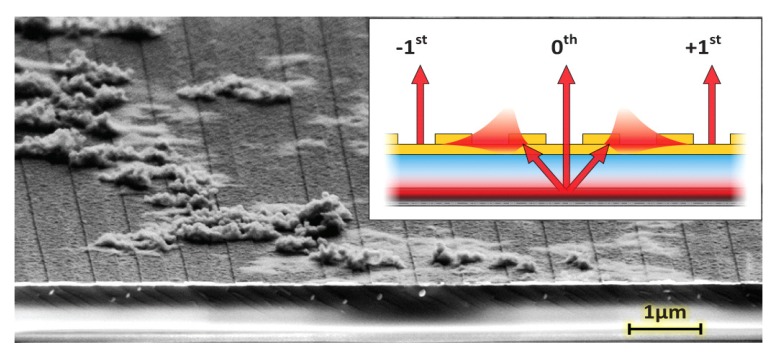
Working principle of the quantum well-surface plasmon resonance (QW-SPR) device. The SEM image presents a grazing angle view of a device sequentially exposed to solutions of neutravidin, biotinylated polyclonal IAV-H3N2 antibodies and capsids of IAV-H3N2. The Au grating is visible on the surface, under which a SiO_2_ layer is deposited atop the GaAs-AlGaAs QW. Inset: Broadband and uncollimated NIR light is emitted from the QW to couple a continuum of SP modes on the surface, since conditions for SPR are concurrently met for a range of E(k_ll_). The propagating modes can then diffract, mainly through the ±1st orders, to be measured by the microscope [9].

**Figure 2:**
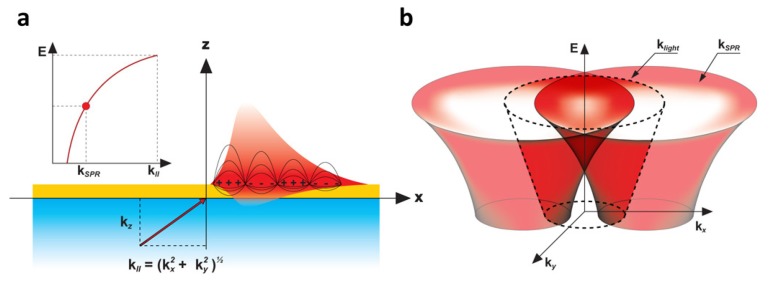
(**a**) Broadband uncollimated induction of SPR. At a given energy, E, and specific wavevector k_ll_ = k_SPR_, electromagnetic charge fluctuations in the form of surface plasmons can be coupled at a metal-dielectric interface. Changing the input energy will modify the required k_ll_ to couple a resonance. As such, there is a continuous dispersion relation E(k_SPR_) for the resonant coupling of the surface plasmons (inset); (**b**) Since k_ll_ can take any direction (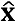, 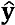) on the surface, the SPR dispersion in the E(k_x_, k_y_) space generates a cone-like shape surface. Note that under normal circumstances k_SPR_ > k_light_. This SPR dispersion is thus scattered within the light cone by the surface corrugation, through the ±1st diffraction order of a grating for example, to be measured by a microscope in the far field [9,13].

An integration of the SPR technology with a light emitting semiconductor is achieved by constructing a metal-dielectric interface atop the solid-state light emitting substrate [2,6,7,8], as shown in Figure 1 [9]. Through electroluminescence or photoluminescence (PL), the embedded quantum well (QW) is excited to emit radiation. For the most general case presented here, the emission is uncollimated (*i.e.*, propagating in all directions) and the broadband spectral bandwidth exceeds 140 nm (1.38–1.65 eV). A dielectric spacer and metal layer are deposited by evaporation on the structure, thus enabling the support of surface plasmon (SP) modes. The metal layer is corrugated in order to extract the SPs into the far field for imaging by a microscope. This top layer can also be biofunctionalized in various manners for specific experiments. The principle behind uncollimated and broadband SP coupling is detailed in Figure 2 [9]. A continuous range of incoming radiation from the QW encounters the metal layer at the dielectric interface.

In Figure 2(a), when the energy (E) and projected wavevectors (|**k_ll_**|) of the incoming light meet specific conditions in E and |**k_ll_**|, a resonance occurs with the surface electrons inducing SPR [2]. It is important to note that such surface charge fluctuations can be coupled at any energy, through their corresponding |k_ll_| (see inset in Figure 2(a)). Moreover, k_ll_ is composed of the two planar directions in 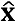 and 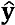. Therefore, at a given energy, SPR can be induced for all the k_ll_ meeting the condition 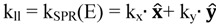. This is shown in Figure 2(b), where the device from Figure 1 supports a continuum of SP modes, whose dispersion relation in E(k_x_,k_y_) is given by a cone-like surface. Because of the diffraction by the surface corrugation, the dispersion relation is duplicated within the measurable light cone of a microscope. To understand such a system in detail, and to be able to design various architectures for specific applications, we have developed a tensorial version of the rigorous coupled wave analysis (TRCWA) proposed by Glytsis and Gaylord [10] and later optimized by many authors [11,12]. This tool enabled the complete customization of the integrated structures required for specific applications; in this case the far field extraction of uncollimated and broadband SPR. A commercial hyperspectral microscope (Photon Etc. Inc., Montreal, QC, Canada) has been set up to study the behavior of our QW-SPR system and to image k_x_-k_y_ directly in the Fourier plane [9,13]. 

### 2.2. Experimental Procedures

**Figure 3:**
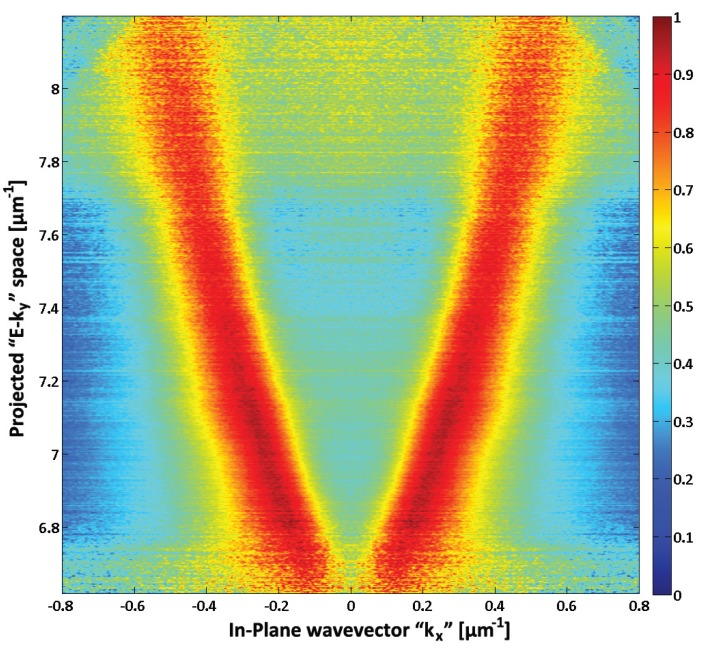
The dispersion relation of the SPR is directly projected on the microscope. The result shows a clear diffraction of SPs, from the ±1st diffraction orders. Measuring the distance between the resonances provides direct information on the surface conditions. In this case, the quality of the signal as a function of E-k_y_ depends on the luminescence intensity of the QW [9,13].

The SPs are diffracted by the surface corrugation and projected in the far field of the microscope. The image shown in Figure 3 presents the measured photonic intensity distribution as a function of projected wavevector **k_x_** in the **E-k_y_** plane [13]. The peak intensities correspond directly to a cross-section of the SPR dispersion relation. From this, the SPR shifts can be monitored by tracking the distances between the two curves, in **k_x_**, as a function of the surface biochemical events. The quantification of the biodetection capabilities of this architecture under real conditions was firstly accomplished using 2% bovine serum albumin (BSA) in phosphate buffered saline (PBS) solutions. Secondly, we examined a specific immobilization of the gamma radiation inactivated H3N2 influenza strain. These viral capsids were trapped with an architecture consisting of a layer of biotinylated polyclonal IAV-H3N2 antibodies attached to neutravidin physisorbed on the Au surface. For the specific immobilization of IAV H3N2, a 200 µg/mL neutravidin solution in PBS was injected on the substrate surface, left to physisorb for 160 min and rinsed with the PBS buffer. A 100 µg/mL solution of biotinylated polyclonal IAV H3N2 antibodies was then injected and left to react with the neutravidin for 160 min. At this stage, the surface was ready to receive a 40 µg/mL solution containing gamma radiation inactivated IAV. A negative test was carried by introducing the IAV H1N1 strain and measuring its surficial shift, Δ_S_. Then the IAV H3N2 strain, of identical concentration, was injected and Δ_S_ measured for positive identification. For both strains, the solutions were left for 60 min to react with the antibody functionalized Au surface.

For comparison purposes, we carried out benchmarking measurements using a commercial Kretschmann–Raether nanoSPR6 system [14], equipped with a basic prism setup, a 1.91 eV (650 nm) laser and an automated goniometer of a 10^−4^ degree angular resolution.

A well-known physisorption of BSA on Au was used to evaluate both the signal-to-noise ratio (SNR) and resolution of the investigated methods. We defined Δ_B_ as the bulk amplitude of the SPR signal (Δ_SPR_) occurring at the saturation for the investigated solution, and Δ_S_ as the surficial shift, measured after the surface is rinsed with the buffer solution, so only molecules physisorbed on the Au surface contribute to the shift.

## 3. Results and Discussion

For the commercial system, BSA physisorption yielded Δ_B_ = 1,081 × 10^−4^ ± 3 × 10^−4^ µm^−1^ and after the PBS rinse, Δ_s_ = 519 × 10^−4^ ± 3 ×10^−4^ µm^−1^ is found. With the same system, the immobilization of inactivated H1N1 IAV, yielded Δ_s_(H1N1) = −10 × 10^−4^ ± 10 × 10^−4^ µm^−1^. In contrast, the targeted H3N2 strain gave an SPR shift of Δ_s_(H3N2) = 92 × 10^−4^ ± 7 × 10^−4^ µm^−1^. These numbers infer, as expected, a distinct preference for the H3N2 strain over the H1N1. For all the experiments carried out with the nanoSPR system, the signal to noise ratio was found to be SNR = 501 ± 13. The Δ_s_(H1N1) ≠ 0 can be explained by the use of IAV H3N2 polyclonal antibodies of broader affinity than their monoclonal counterpart and possibly by an imperfect surface coverage with the physisorbed neutravidin complex, leaving some Au regions exposed to H1N1 capsids. Specificity was further confirmed through fluorescence and scanning electron microscope (SEM) imaging. On average, 5 capsids/µm^2^ were observed with SEM for the samples exposed to H3N2. The results demonstrate reliable immobilization dynamics and they offer a comparison benchmark concerning the immobilization of BSA molecules and IAV H3N2 virus, on which the evaluation of the QW-SPR platform can be established. 

**Figure 4:**
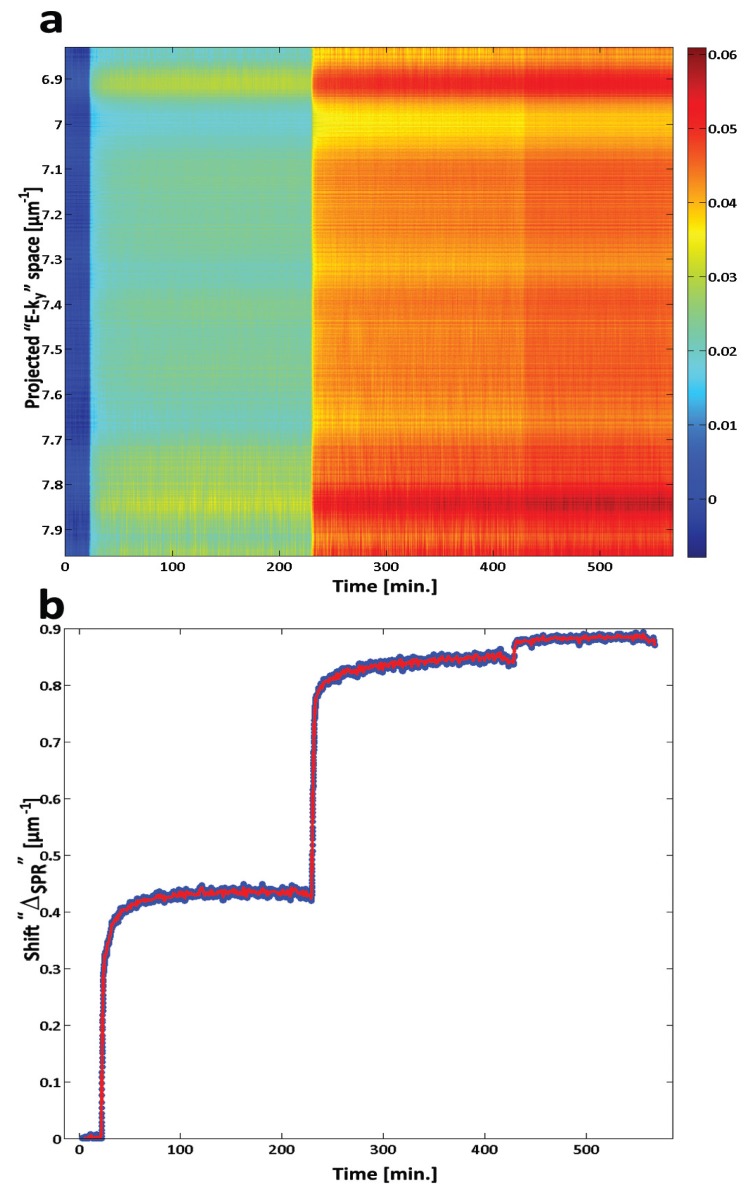
Detection of H3N2 IAV with a QW-SPR device. (**a**) Δ_SPR_ as a function of E-k_y_ and time, as the neutravidin (0 < t ≤ 230 min), biotinylated polyclonal IAV H3N2 antibodies (240 < t ≤ 420 min) and inactivated IAV H3N2 (t > 420 min) solutions are injected over the device and rinsed. (**b**) Cumulative SPR shift, Δ_SPR_|_Total_, in time for the IAV H3N2 adsorption. A surficial shift of Δ_s_ = 33 × 10^−3^ ± 1 × 10^−3^ µm^−1^ is measured after rinsing the surfaces with PBS [9].

Figure 3 shows a single frame (2.2 s collection time) result obtained with our approach during physisorption of BSA on the surface of a QW-SPR device [9]. The diffracted SP modes, represented by fragments of two ellipses, are clearly visible. Note that the quality of the signal as a function of E-**k_y_** depends on the luminescence intensity of the QW, which here is manifested by the increased noise amplitude of the top and bottom parts of the recorded frame. The information on the SPR conditions on the surface of the QW-SPR device can be obtained by measuring the distance between the ellipses as a function of time. In the case of a broadband and uncollimated SPR system, the shift occurs in E, **k_x_** and **k_y_**. Therefore, a cumulative shift between the two SPR peak surfaces corresponds to Δ_SPR_|_Total_= {℘ Δ_SPR_ |E-**k_y_**|^2^}^½^. For BSA physisorption, we measured Δ_s_ = 7,140 × 10^−4^ ± 60 × 10^−4^ µm^−1^ after the PBS rinse. For the specific immobilization of IAV H3N2, the dynamic variations of Δ_SPR_|_Total_ are presented in Figure 4. Note that the periodic modulations in E-k_y_ (Figure 4(a)) are attributed to the interactions of the SPs with the grating as predicted in the literature [13,15,16]. In this case, the shift induced by the selective immobilization of H3N2 is Δ_s_ = 33 × 10^−3^ ± 1 × 10^−3^ µm^−1^ after the PBS rinse. For this system, the resulting signal to noise ratio was found to be SNR = 1,831 ± 12. When comparing the SNR of the SPR signal generated by the QW-SPR architecture with that of the commercial system, we find the former generates slightly more stable datasets. This is mainly attributed to the continuous surface coupling of the SPR over a wide range of E and **k_ll_**, resulting in the cumulative shifts over the collected dispersions that are greater than the potential shift over a single variable in E or **k_ll_** values. In this hyperspectral acquisition mode, the shot noise of the system is thus reduced by upsampling the number of monitored SPR events (in E and **k_ll_** in time).

To address the SPR signal resolution issue, we investigated the response of the commercial nanSPR6 and QW-SPR systems to a 20 mg/mL solution of BSA in PBS (0.3 µM). Literature [17] shows that the Au surface saturates with BSA following 36 h exposure at a 1.5 µM BSA solution, which yields a mass coverage of 1.976 ng of BSA per 1 mm^2^ (ρ_BSA_ = 1.36 × 10^6^ g/m^3^ for 1.45 nm tall molecules). A weaker BSA concentration of 450 pM leads to coverage of 0.75 ng/mm^2^ [18]. Using these results, we estimated that our samples (at 0.3 µM) contained a surficial coverage of 0.9949 ng/mm^2^, *i.e.*, approximately 50% of the surface was covered with BSA. Using the Gladstone-Dale formula [19], we estimated the specific refractive index increment of the 0.3 μM (20 mg/mL) BSA solution in PBS to be equal to 0.1845 cm^3^/g × 20 × 10^−3^ g/mL = 0.0037 RIU. Furthermore, the Barer and Akimoto data [20,21] allow to determine the refractive index of 0.3 µM BSA in solution as n_BSA2%_ = 1.3369 @ 650 and 1.3330 @ 870 nm (n_PBS_ = 1.3332 @ 650 nm and 1.3293 @ 870 nm).

From [22,23] and the product information at Sigma-Aldrich, we can assume that the average height of a continuous BSA film is 1.45 nm. Also, wavelength dependence of the refractive index of a monolayer of BSA in the VIS-NIR region can be de described as n_BSA_(λ) = 1.563 + 3,505·λ^−1^, with λ in nm [19,20]. Therefore, after rinsing the BSA covered surface at 0.9949 ng/mm^2^, the SP evanescent tail is exposed to a layer of BSA approximately 1.45 nm thick (n_BSA_ = 1.4531 at 650 nm and n_BSA_ = 1.4493 at 870 nm), while the remaining environment is provided by PBS. To evaluate the surficial effective shift in refractive index, we take:

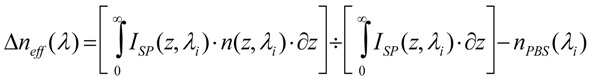
(1)

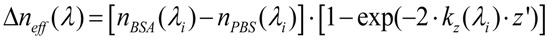
(2)
where I_SP_ (z,λ_i_) = |E|^2^ is the E-field intensity in **z**, n(z, λ_i_) is a spatially dependent refractive index, z′ is the height of BSA, and λ_i_ denotes 650 or 870 nm. For the nanoSPR6, this yields a Δn_eff_ = 16.75 × 10^−4^ at 650 nm. Therefore, Δ_B_ = (1,081 ± 3) × 10^−4^ µm^−1^ for a 0.0037 RIU shift corresponds to 29.22 µm^−1^/RIU sensitivity in “bulk” and 1.03 × 10^−5^ RIU resolution (NanoSPR Inc. claims 2 × 10^−5^ RIU sensitivity). The surface sensitivity Δ_s_ = (519 ± 3) × 10^−4^ µm^−1^ for 16.75 × 10^−4^ RIU shift yields 149.85 µm^−1^/RIU surficial sensitivity and 9.68 × 10^−6^ RIU in resolution.

In the same manner, for the QW-SPR conic dispersion, we have Δ_B_ = (7,569 ± 7) × 10^−4^ µm^−1^ for 0.0037, *i.e.*, a 204.56 µm^−1^/RIU sensitivity in “bulk” or 3.42 × 10^−6^ RIU resolution. The surface sensitivity is given by a Δ_s_ = (7,140 ± 6) × 10^−4^ µm^−1^ for 10.39 × 10^−4^ RIU shift, *i.e.*, a 7,099 µm^−1^/RIU surficial sensitivity of 1.45 × 10^−6^ RIU resolution. A summary of the values involved in this analysis is presented in Table 1. 

**Table 1:** Summary of refractive indices and sensitivities.

Variable	Value
Surface coverage of physisorbed BSA (0.3 µM) after PBS rinse [17,18]	0.9949 ng/mm^2^
Refractive index shift from 0.3 µM BSA in PBS [19,20,21]	0.0037 RIU @ 650 nm and 870 nm
Refractive index of PBS [19,20,21]	1.3332 @ 650 nm | 1.3293 @ 870 nm
Refractive index of BSA monolayer (0.9949ng/mm^2^)	1.4531 @ 650 nm | 1.4493 @ 870 nm
Average height of BSA [22,23] and Sigma-Aldrich	1.45 nm
Effective refractive index shift after rinsing (Δn_eff_)	16.75 × 10^−4^ @ 650 nm | 10.39℘10^−4^ @ 870 nm
Signal to Noise ratio NanoSPR6 (650 nm)	501 ± 13
Signal to Noise ratio QW-SPR Conical (870 nm)	1,831 ± 12
Surface sensitivities and resolutions for NanoSPR6 (650 nm) [13]	Δ_S_ = 149.9 µm^−1^/RIURes = 9.68℘10^−6^ RIU
Surface sensitivities and resolutions for QW-SPR Conical (870 nm) [13]	Δ_S_ = 7,099 µm^−1^/RIURes = 1.45℘10^−6^ RIU

The surprising high sensitivity of the QW-SPR method, comparable to the best values reported in the SPR literature [24,25] could be explained by an optimal trade-off between an attractive dynamic range and a stable signal, with many points in time (SNR). A greater luminous flux from the QW source would yield better SNR. In addition, as we take advantage of broadband SPR coupling for the improvement of the SNR factor (by upsampling), a larger luminescence bandwidth should yield even better sensitivities with this approach. Of particular interest for biosensing applications is the excellent time resolution (2.2 s) of the QW-SPR method. 

## 4. Conclusions

Interfacing a sensitive SPR transducer with a µ-TAS system would enable a dissemination of this characterization method into numerous fields of applications that currently cannot afford the space, capital or trained personnel to carry out complete experiments. A potentially efficient way to integrate SPR into a micro-system is through the use of existing semiconductor technology, where an integrated light source and transducer can be part of a single nanosystem embedded with relevant microelectronic circuits. We have been exploring this approach and presented here results that reviewed the recent progress we achieved in that context. The integration of a QW emitter with the Au surface (QW-SPR) that could be functionalized using conventional biochemistry enabled us to achieve an ultimate miniaturization of the device. The analysis of the results obtained with this innovative approach is accomplished through the conjugate field of a microscope that, as a result of the simultaneous inclusion of resonances coming from a wide spectrum of energies and wavevectors, offers attractive conditions for sensitive measurements. We demonstrate that the application of a conical mode of collecting SPR data from a QW-SPR device produces the results (SPR scan) in less than 2.2 s, while the resolution of the method approaches 1.45 × 10^−6^ RIU, *i.e.*, it is comparable with the best results reported to date in the SPR literature. In contrast with conventional methods of monitoring SPR signals requiring dedicated systems, SPR tracking through microscopy is a more practical and affordable approach of wider applicability. We have demonstrated the performance of our semiconductor-SPR biosensing device for monitoring simple biochemical reactions and detection of the influenza A virus. This is a significant milestone in the development of a family of innovative devices designed for remote biosensing. The QW-SPR technology makes it possible to interface the SPR platform with traditional microelectronics and, thus, it has the potential to open a wide range of new applications that go beyond the traditional field of biosensing. 
